# Short-Term Arrhythmia Prediction Using AI Based on Daily Data From Implantable Devices: Multicenter Prospective Observational Study

**DOI:** 10.2196/85841

**Published:** 2026-03-18

**Authors:** Ignacio Fernández Lozano, Joaquín Fernández de la Concha, Javier Ramos Maqueda, Nicasio Pérez Castellano, Rafael Salguero Bodes, F Javier García-Fernández, Juan Benezet Mazuecos, Javier Jiménez Candil, Tomás Datino, Sem Briongos Figuero, Javier Paniagua Olmedillas, Miguel Nicolás Font de la Fuente, Juan López-Dóriga Costales, Sarai Paz Fernández, Vicente Copoví Lucas

**Affiliations:** 1 Heart Disease Institute, Hospital Universitario Puerta de Hierro Majadahonda, C. Joaquín Rodrigo, 1, Majadahonda, Madrid, 28222, Spain, 34 911 91 60 00; 2 Heart Disease Institute, Hospital Universitario de Badajoz, Badajoz, Spain; 3 Heart Disease Institute, Hospital Clínico Universitario Lozano Blesa, Zaragoza, Spain; 4 Heart Disease Institute, Clínica HLA Montpellier, Zaragoza, Spain; 5 Heart Disease Institute, Hospital Clínico San Carlos, Madrid, Spain; 6 Heart Disease Institute, Hospital Universitario 12 de Octubre, Madrid, Spain; 7 Heart Disease Institute, Hospital Universitario de Burgos, Burgos, Spain; 8 Heart Disease Institute, Hospital La Luz, Madrid, Spain; 9 Heart Disease Institute, Complejo Hospitalario de Salamanca, Salamanca, Spain; 10 Heart Disease Institute, Hospital Universitario Quirónsalud Madrid, Madrid, Spain; 11 Heart Disease Institute, Hospital Universitario Infanta Leonor, Madrid, Spain; 12 Heart Disease Institute, Hospital Virgen de la Concha, Zamora, Spain; 13 Monitoring Life S.A, Santa Cruz de Tenerife, Spain; 14 Arrhythmia Network Technology SL, Madrid, Spain

**Keywords:** artificial intelligence, AI, atrial fibrillation, AF, machine learning, telemedicine, pacemaker, arrhythmia prediction, predictive medicine

## Abstract

**Background:**

Predictive medicine relies on algorithms to determine clinical treatments tailored to each patient’s individual characteristics. Predictive models based on artificial intelligence have shown promise in identifying atrial fibrillation episodes; however, they rarely focus on short-term dynamic prediction.

**Objective:**

This study aimed to evaluate the use of an artificial intelligence model and remote monitoring data extracted from pacemaker devices to predict the onset or worsening of arrhythmias in the short term.

**Methods:**

This was a multicenter prospective observational study in which data from 314 patients were analyzed. A total of 65,243 data sequences were collected, of which 55,532 (85.1%) were used to train the algorithm. This model used 31-day records to predict whether the number of arrhythmic episodes would increase, decrease, or remain the same in the following 14 days.

**Results:**

The sensitivity and specificity of the generated predictions were calculated from 9711 prediction-observation pairs. The global sensitivity was 66.4% (95% CI 64.3%-68.3%), and specificity was 77.4% (95% CI 76.4%-78.4%). For patients with baseline arrhythmia, sensitivity was 76.8% (95% CI 74.6%-78.8%), and specificity was 39.6% (95% CI 35.8%-43.5%). The prediction for patients with no baseline arrhythmia showed a sensitivity of 39% (95% CI 35.1%-43%) and a specificity of 81% (95% CI 80.0%-81.9%). The analysis for the patient subgroup without history of atrial fibrillation (232/314, 73.9%) yielded a 69% sensitivity (95% CI 66.5%-71.5%) and an 80% specificity (95% CI 79.3%-81.3%).

**Conclusions:**

This model was capable of predicting short-term increases or decreases in arrhythmic episodes with reasonable sensitivity and specificity using data collected through remote monitoring of implantable devices. The model’s performance is expected to improve progressively as more data samples become available, including demographic data and clinical records.

## Introduction

In recent years, the use of artificial intelligence (AI) for the diagnosis, prognosis, and evaluation of risk factors and the response to treatment of different pathologies has shown significant advancements. This has had a positive impact on health care systems, improving the efficiency of medical handling and reducing the number of adverse events [1,2].

Cardiac electrophysiology has emerged as one of the fastest-growing subspecialties within cardiology over the last few decades [3,4], which is evidenced by numerous technological advancements aimed at detecting, predicting, and managing cardiac arrhythmias [5,6]. Among those, the prediction of cardiac arrhythmias is especially relevant for patients with atrial fibrillation (AF), who are 5 times more likely to experience thromboembolic events than patients without AF [7].

AI training of information systems based on enormous data quantities from clinical reports and complementary tests from millions of patients not only enables episode prediction in patients diagnosed with AF but also allows for the diagnosis of asymptomatic patients, allowing for early intervention and reducing the number of clinical events [8]. However, cardiac implantable devices can also be leveraged to train AI models for the detection of cardiac arrhythmias. They are particularly well suited for machine learning (ML)–based arrhythmia prediction because they provide continuous, high-fidelity rhythm monitoring over long periods, enabling precise quantification of atrial arrhythmia burden and enhancing diagnostic remote monitoring workflow and device-based therapies [9], with the additional advantage of being already implanted on the patient.

Although several authors have published many AI- and ML-based models for AF prediction in different contexts with good results [10-12], these models were primarily trained and evaluated for predictions over extended periods (often up to 1 year), which provided them with a great amount of input data. However, algorithms supporting short-term, dynamic AF prediction in patients with implantable cardiac devices are scarce. Short-term arrhythmia prediction is uniquely challenging because it requires anticipating nonlinear state transitions driven by heterogeneous and rapidly evolving physiological signals. While long-term prediction is valuable for estimating the risk of AF development in the general population—supporting primary prevention and population-level care planning—dynamic short-term prediction may be particularly impactful in patients who have already been diagnosed with AF. In this context, continuously collected data from implantable cardiac devices enable high-resolution, longitudinal assessment of cardiac rhythm, creating an opportunity for near-term forecasting of AF burden progression. Such device-based prediction could support timely optimization of medical therapy, generation of real-time alerts, and prevention of downstream adverse outcomes. Moreover, in patients with newly diagnosed AF, device-derived dynamic prediction may facilitate closer disease monitoring, guide medication escalation, and enable proactive risk factor modification [10,13].

For that reason, this study aimed to evaluate the use of AI on data extracted from remote pacemaker monitoring for short-term prediction of the onset or increase of AF episodes. Our hypothesis was that data obtained from remote pacemakers could be used to train AI models to predict the onset of atrial arrhythmias with a relatively high sensitivity and specificity without the need for additional datasets.

## Methods

### Study Design

IA Pacing is a multicenter prospective observational study that started in May 2022 and is ongoing as of January 2026 across 12 centers in Spain. All participating centers used implantable cardiac pacing devices from the same manufacturer (Boston Scientific) with no variation in the telemetry or remote monitoring platform, which was uniformly provided by the vendor. Remote monitoring procedures consisted of daily monitoring.

### Ethical Considerations

All procedures performed in this study were conducted in compliance with relevant laws and institutional guidelines and were approved by the ethics committees of the participating centers, including the Comité Ético de Investigación Clínica del Área de Zamora (approved May 25, 2023), Comité Ético de la Investigación Médica of Hospital Universitario 12 de Octubre, Comité Ético de Investigación Clínica de Badajoz (updated November 30, 2021; protocol code: IA-Pacing), Comité Ético de la Investigación Clínica of Hospital Universitario Clínico San Carlos, Comité Ético de la Investigación con Medicamentos of Hospital Universitario Infanta Leonor, Hospital Lozano Blesa (under the protocol approved by the Comité Ético de la Investigación con Medicamentos of Hospital Universitario Puerta de Hierro), Clínica Quirón La Luz and Quirón Pozuelo (both contingent upon favorable review by the Comité Ético de la Investigación con Medicamentos of Hospital Universitario Puerta de Hierro), and Comité Ético de Investigación Médica del Área de Salud de Salamanca (approved March 30, 2022).

Written informed consent was obtained from all participants before enrollment in the study. Participants were informed about the objectives and procedures of the study and their right to withdraw at any time without consequences for their medical care. All procedures performed in this study were conducted in accordance with the ethical principles of the Declaration of Helsinki and complied with applicable European and Spanish regulations governing biomedical research and personal data protection, including the General Data Protection Regulation (Regulation [EU] 2016/679), Ley 14/2007 de Investigación Biomédica, and Ley Orgánica 3/2018 de Protección de Datos Personales y garantía de los derechos digitales.

To protect participant privacy and confidentiality, all data were pseudonymized before analysis. Identifiable information was stored separately from the research dataset in secure institutional systems with restricted access, and only authorized members of the research team were allowed to access the data. Data handling and storage complied with institutional data protection policies and applicable European and Spanish data protection regulations.

Participants did not receive financial compensation for their participation in the study. Inclusion criteria for the study and complete patient recruitment by hospital can be found in Multimedia Appendix 1.

### Baseline Characteristics of the Population

Medical records were used to assess patients’ baseline characteristics, including demographic data, cardiovascular risk factors, history of arrhythmias, details of the implanted device, procedural data, and pharmacological treatment.

### Prediction of Arrhythmia Onset Using AI

To calculate arrhythmia predictions, we used data generated through remote monitoring of patients’ pacemakers, which included the following variables:

- General variables—activity level, respiratory rate, and maximum and mean heart rate
- AF-related variables—total number of arrhythmia episodes per day, total duration of those arrhythmias, atrial tachycardia response (mean and maximum ventricular rate during rapid ventricular response events), and apnea-hypopnea index
- Heart failure–related variables—percentage of left ventricular pacing and SD of the average NN intervals every 5 minutes

When considering arrhythmia episodes per day, we took into account only those arrhythmias that were of the “supraventricular tachycardia” or “atrial fibrillation” type as categorized by the device, with no time limit restrictions.

The algorithm divided the data into sequences of 45 days. The first 31 days were used for the algorithm to analyze and predict what would happen in the following 14 days. This time was selected after various tests with different times of training and prediction, with no major changes among them. Furthermore, based on medical criteria, a 14-day predictive time window was considered sufficient to allow for patient scheduling and timely intervention if required. Therefore, after observing the telemetry data for 31 days, a prediction was generated for the following 14 days. The appearance or absence of arrhythmias was divided into 4 categories as follows:

- Increase—the patient already had arrhythmias during the 31 days of observation, and the average number of arrhythmias during the 14 days of prediction increased.
- Decrease—the patient already had arrhythmias during the 31 days of observation, but the average number of arrhythmias during the 14 days of prediction decreased.
- Remains at 0—the patient did not have arrhythmias during the 31 days of observation, and the average number of arrhythmias during the 14 days of prediction remained at 0.
- Goes from 0 to something—the patient did not have arrhythmias during the 31 days of observation, but some arrhythmias occurred during the 14 days of prediction.

Subsequently, to simplify the analysis, the previous 4 categories were regrouped into 2:

- Increased—the average number of arrhythmias during the day increased (categories 1 and 4).
- Maintained or decreased—the average number of arrhythmias during the day was maintained or decreased (categories 2 and 3).

Detailed information about these categories is provided in Multimedia Appendix 2. Similarly, information about data preprocessing and final data volume is provided in Multimedia Appendix 3. Finally, the creation of the predictive model using AI, along with its parameters, its training, and its final results over the *test* data, is detailed in Multimedia Appendix 4.

Furthermore, to generate explanations in the model, a specialized tool called Shapley additive explanations (SHAP) was used, which leverages game theory–based results to identify the variables that had the greatest importance in making each prediction. In Multimedia Appendix 5, the average results for the predictions of each class can be observed, where some clinically relevant variables stand out, such as the presence of apneas or the SD of the average NN intervals. Alongside SHAP, the entropy of the classification was used as a measure of uncertainty to obtain confidence estimations for each prediction, as explained in Multimedia Appendix 6. These tools combined reduce the opacity of AI models, increasing trust in them as a tool for clinical decision-making.

### Web Application Development

On the basis of the AI-generated predictions of arrhythmias for the following 14 days and the explanations based on SHAP and confidence estimation, a web application was developed to allow medical personnel to monitor each patient.

### Statistical Analysis

Categorical variables are presented as frequencies and percentages, whereas quantitative variables are expressed as means and SDs. The sensitivity and specificity of the arrhythmia prediction were calculated from a 2 × 2 contingency table considering prediction and reality during the follow-up period. Sensitivity and specificity were calculated from 9711 prediction-observation pairs. Statistical analyses were performed using SPSS (version 17.0; IBM Corp).

## Results

Data from 314 patients enrolled in the IA Pacing study between May 2022 and May 2024 were analyzed. A total of 65,243 data sequences from implantable devices were collected, of which 55,532 (85.1%) were used to train the algorithm, and the remaining 9711 (14.9%) were used to analyze the sensitivity and specificity of the generated predictions.

### Baseline Characteristics of the Population

Table 1 shows the clinical characteristics of the participating patients. A total of 61.3% (192/313) of the patients were male, and the mean age was 77 (SD 9.2) years. The most common indication for pacemaker implantation was atrioventricular block, whereas the predominant pacing site was the right ventricular apex. In total, 81.9% (158/193) of the patients had at least one cardiovascular hospitalization in the year before enrollment.

**Table 1.** Baseline characteristics of the patients (N=314). Variables are reported as n/N to account for missing data.

Baseline characteristics	Values
Sex (male), n/N (%)	192/313 (61.3)
Age (y), mean (SD)	77 (9.2)
Pacemaker indication among patients, n/N (%)	
AV^a^ block	240/313 (76.7)
Sinus bradycardia	24/313 (7.7)
Other indications	49/313 (15.7)
Pacing type among patients, n/N (%)	
Right ventricular apex	259/310 (83.5)
Left branch	51/310 (16.5)
Type of device used by patients, n/N (%)	
Dual-chamber pacemaker	302/314 (96.2)
Tricameral pacemaker with resynchronizer	12/314 (3.8)
Pacemaker models used by patients, n/N (%)	
Boston Scientific PROPONENT MRI L211	101/314 (32.2)
Boston Scientific ACCOLADE MRI L311	67/314 (21.3)
Boston Scientific PROPONENT EL MRI L231	15/314 (4.8)
Boston Scientific ACCOLADE EL MRI L331	119/314 (37.9)
Boston Scientific VISIONIST X4 CRT-P U225	3/314 (1)
Boston Scientific VISIONIST X4 CRT-P U228	9/314 (2.9)
Patients with CV^b^ hospitalizations in the last y, n/N (%)	
0	35/193 (18.1)
1	132/193 (68.4)
*>*2	26/193 (13.5)
CV risk factors among patients, n (%)	
Arterial hypertension	236/308 (76.6)
Diabetes mellitus	118/296 (39.9)
Dyslipidemia	202/302 (66.9)
Obesity	54/273 (19.8)
Current smoking	22/286 (7.7)
Current alcoholism	14/275 (5.1)
CV history among patients, n/N (%)	
NYHA^c^ I	101/187 (54)
Ischemic cardiopathies	52/277 (18.8)
Valvulopathies	77/284 (27.1)
Myocardiopathies	34/279 (12.2)
TIA^d^ or stroke	36/277 (13)
Peripheral vascular disease	21/269 (7.8)
AF^e^ stroke risk score (CHA_2_DS_2_-VASc), mean (SD)	3.5 (1.5)
Arrhythmia history among patients, n/N (%)	
AF	42/275 (15.3)
Ventricular arrhythmia	5/313 (1.6)
Other comorbidities among patients, n/N (%)	
COPD^f^	24/273 (8.8)
Asthma	17/270 (6.3)
Pharmacological treatment among patients, n (%)	
Antiaggregants	124/314 (39.5)
Anticoagulants	57/314 (18.2)
ACE^g^ inhibitors, ARBs^h^, or ARNIs^i^	178/314 (56.7)
β-blockers	79/314 (25.2)
Loop diuretics	66/314 (21.1)

a AV: atrioventricular.

b CV: cardiovascular.

c NYHA: New York Heart Association.

d TIA: transient ischemic attack.

e AF: atrial fibrillation.

f COPD: chronic obstructive pulmonary disease.

g ACE: angiotensin-converting enzyme.

h ARB: angiotensin II receptor blockers.

i ARNI: angiotensin receptor-neprilysin inhibitor.

Regarding cardiovascular risk factors, most patients had hypertension (236/308, 76.6%) and dyslipidemia (202/302, 66.9%), whereas the prevalence of diabetes mellitus was 39.9% (118/296). The mean CHA_2_DS_2_-VASc score was 3.5 (SD 1.5). A history of AF was present in 15.3% (42/274) of the patients.

### Prediction of Arrhythmia Onset Using AI

The sensitivity of the AI-based predictive model was 66.4% for the full sample (1456 true positive cases; 1696 false positive cases; 95% CI 64.3%‐68.3%), whereas specificity was 77.4% (95% CI 76.4%‐78.4%), corresponding to 738 false negative cases and 5821 true negative cases.

Next, we grouped the patients based on whether they had presented arrhythmias during the initial 31-day period used by the algorithm to generate predictions. Two groups were defined: patients with arrhythmias during the baseline period (those classified in the “increase” or “decrease” categories) and patients without arrhythmias during the baseline period (those in the “remains at 0” or “goes from 0 to something” categories).

On the basis of the contingency tables generated, we obtained a sensitivity of 76.8% (95% CI 74.6%‐78.8%) and specificity of 39.6% (95% CI 35.8%‐43.5%) in patients with arrhythmias during the baseline period (1219 true positive cases, 393 false positive cases, 258 true negative cases, and 368 false negative cases) and a sensitivity of 39% (95% CI 35.1%‐43%) and specificity of 81% (95% CI 80%‐81.9%) in patients without arrhythmias during that period (237 true positive cases, 1303 false positive cases, 370 false negative cases, and 5563 true negative cases).

In addition, using data sequences from patients with no history of AF (232/314, 73.9%; 7259 data points), we calculated the model’s sensitivity and specificity for arrhythmia prediction considering both supraventricular tachycardia and AF as the presence of arrhythmia. These were 69% (95% CI 66.5%‐71.5%) and 80% (95% CI 79.3%‐81.3%), respectively.

The raw values from the contingency table for each group can be found in Table 2.

**Table 2.** Arrhythmic episode prediction and ground truth for both patient groups.

AI^a^ arrhythmia prediction	Follow-up ground truth		Sensitivity (%)	Specificity (%)
	Increased	Maintained or decreased		
Total patients, n			66.4	77.4
Increased	1456	1696		
Maintained or decreased	738	5821		
Patients with arrhythmia in the last 31 days, n			76.8	39.6
Increased	1219	393		
Maintained or decreased	368	258		
Patients without arrhythmia in the last 31 days, n			39.0	81.2
Increased	237	1303		
Maintained or decreased	370	5563		
Patients with no AF^b^ history (n=232), n			69.1	80.3
Increased	951	1157		
Maintained or decreased	426	4725		

a AI: artificial intelligence.

b AF: atrial fibrillation.

### Web Application Development

**Figure 1.**
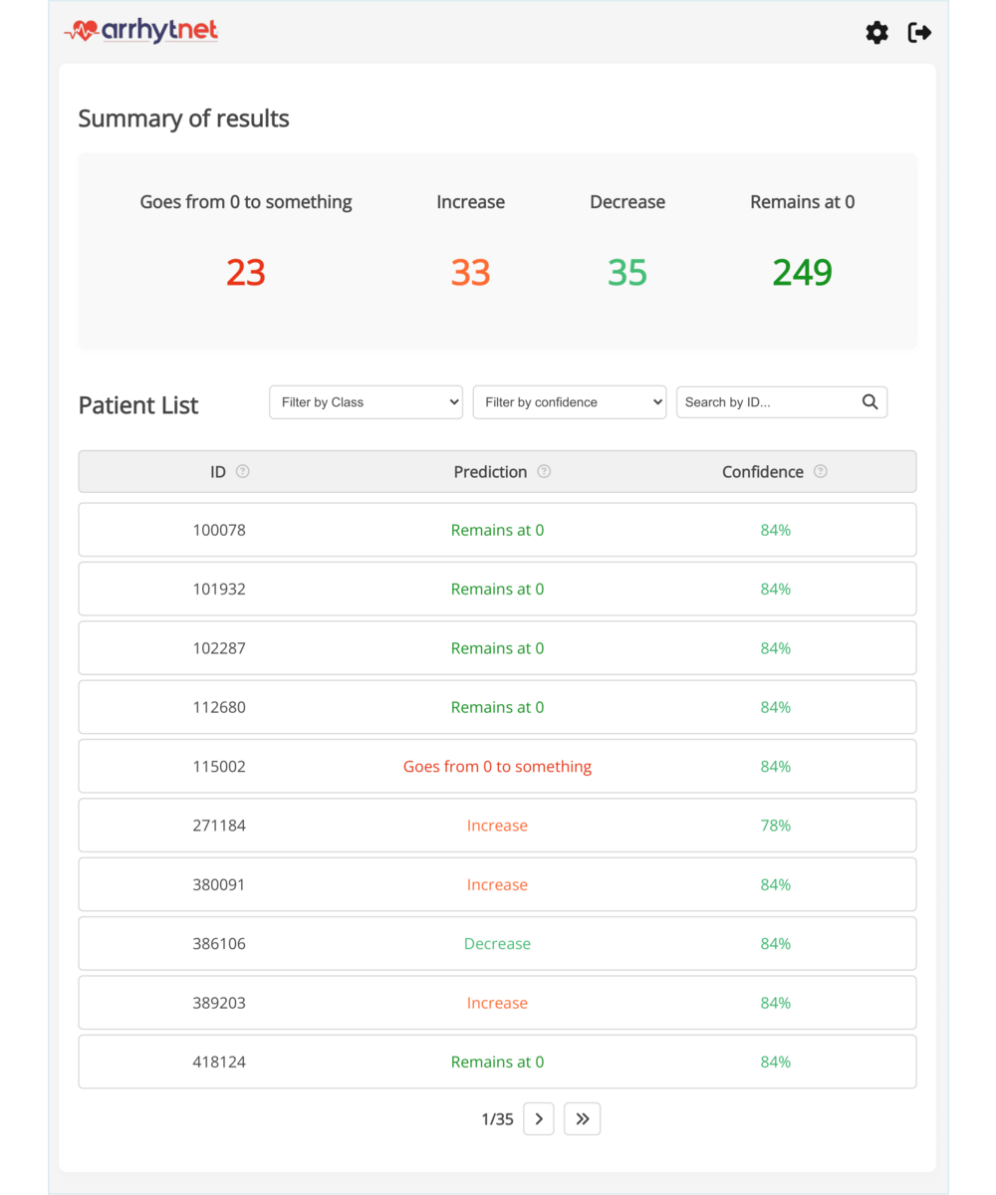
Main view of the web platform showing the list of patients with their predictions and estimated confidence along with a summary of the patients’ results and search filters to aid physicians (the number of patients is higher as predictions can be made on patients with 31-45 days of measurement instead of just the >45 days used to validate the artificial intelligence model).

**Figure 2.**
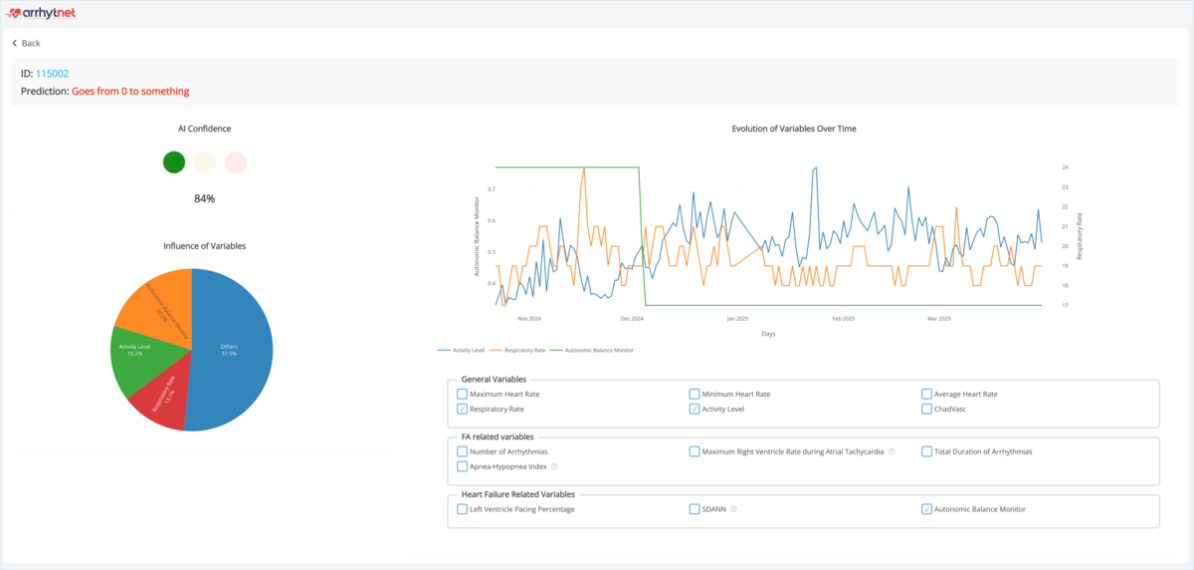
Details of a patient with their prediction, estimated confidence, most important variables for decision-making based on Shapley additive explanations (pie chart), and variable evolution.

The web application interface can be observed in Figure 1 and Figure 2 and presents the prediction along with its confidence score (expressed as a percentage), aiding clinical decision-making. The main view of the web platform shows the list of patients with their predictions and estimated confidence along with a summary of the patients’ results and search filters to aid physicians (the number of patients is higher as predictions can be made on patients with 31-45 days of measurement instead of just the >45 days used to validate the AI model). Details of a patient with their prediction, estimated confidence, most important variables for decision-making based on SHAP (pie chart), and variable evolution can also be observed on the web platform.

## Discussion

AI implementation is transforming health care in cardiology, especially in the context of heart rhythm–related disorders. We developed an initial algorithm with information obtained from the remote monitoring of pacemakers that predicted atrial arrhythmia onset within the 14 days following the 31-day monitoring period with a 66.4% sensitivity and 77.4% specificity. This study is, to the best of our knowledge, the first to use data exclusively from remote pacemaker monitoring to dynamically predict arrhythmia onset in patients with atrial arrhythmia in the short term.

Given how important arrhythmia detection is for the identification of patients at risk of having a stroke or systemic embolism, AI algorithms have lately been used for detection, prediction, and treatment tailoring of patients with AF [8]. Focusing on AF episode prediction, several authors have achieved significant advancements in developing AF-predicting algorithms in varying periods from initial measured data. Kao et al [14] developed a model able to predict new arrhythmic episodes in the span of a year with a 98.7% specificity using a database of clinical data gathered for 3 years from older patients with no electrocardiographic data. Similarly, Tiwari et al [15] predicted 6-month AF onset with 75% sensitivity and 85% specificity by developing a model using 200 clinical features from more than 2 million clinical records. Another algorithm worth mentioning developed from clinical records is the one from the Future Innovations in Novel Detection of Atrial Fibrillation study by Nadarajah et al [16] that predicted AF onset within 6 months with a sensitivity and specificity of 78.1% and 73.1%, respectively. Clinical data have also been used to train ML models to detect risk factors for developing AF in asymptomatic patients, as described by Hill et al [10]. Another author used data from 100,000 electrocardiograms (ECGs) taken from 40,000 patients in a mean of 2 years of follow-up to predict the time before a new arrhythmic episode, obtaining similar results to medical record–based predictions [11]. In our study, unlike the studies cited before, no medical record was used to train the system. The algorithm compared daily data from the remote monitoring of an implantable device, similarly to the recently published study by Gregoire et al [13] that trained an ML model using Holter recordings to predict short-term AF episodes. We consider this a strength of our study as only data obtained from the remote monitoring of the implantable device were needed to estimate the risk of experiencing an arrhythmic episode, thus eliminating the need for additional information from medical records.

Although our algorithm does not compute the time before a new arrhythmic episode, it does enable the identification of patients at risk of experiencing episodes of supraventricular arrhythmias in the short term (14 days following the measurement), which would improve their medical care. Our model correctly predicted whether arrhythmias would increase in almost 75% of the cases, with 66.4% global sensitivity and 77.4% specificity. The previously mentioned study from Gregoire et al [13] predicted short-term paroxysmal AF episode onset with higher sensitivity and specificity than ours (86.6% vs 83.0%, respectively). However, it is worth mentioning that, while their approach relies on comprehensive ECG recordings from a Holter monitor, our analysis is based solely on limited, secondary data derived from pacemaker use (eg, daytime heart rate, respiratory rate, or apnea counts). Although these variables provide less information, they may still be highly informative as they are known to be correlated with arrhythmic episodes [17]. Additionally, it is noteworthy that our algorithm was trained using data obtained from a device already implanted in the patient, thereby eliminating the need for additional medical visits. A table comparing the main features of our study to those of the ones mentioned previously can be found in Multimedia Appendix 7.

In patients with no registered history of arrhythmia (232/314, 73.9%), who constitute the group most likely to benefit from early therapeutic interventions, the analysis showed higher values of sensitivity and specificity (69% and 80%, respectively). Algorithm performance was different according to baseline arrhythmia status (higher sensitivity in patients with arrhythmia in the last 31 days and higher specificity in patients with no episodes in the same period). We think this is due to the different data distribution among these 2 groups as sensitivity and specificity heavily depend on the prevalence of the episode of interest (in this case, arrhythmia).

The fact that our algorithm predicted the occurrence of arrhythmias with a similar performance to that of the model by Tiwari et al [15] (which predicted arrhythmias in a 6-month time window based on clinical records from 2 million younger patients) with relatively few data is a promising starting point to continue enhancing AI training and, therefore, the obtained results. Similarly to Tiwari et al [15], we deem it necessary to optimize AI models to increase their sensitivity and reduce false positives. One strength of our approach is that it requires only a small amount of data for training because the implantable devices continuously monitor cardiac activity. We also used a tool to standardize variable names across devices, which allowed us to combine datasets and increase the amount of data for training, which is another strong point of this study.

It is worth mentioning some limitations of the proposed model. First, the training did not consider demographic variables. Including these factors could increase the predictive capabilities and justify the use of AI over classic risk factor predictors [18]. Moreover, it has been observed that models encompassing more than one type of variable—such as clinical data and ECGs, echocardiograms, or magnetic resonance imaging, for instance—increase prediction sensitivity [19]; this should be taken into account when designing and training future AI algorithms. Second, although the number of prediction and reality sequences was high (more than 9000), data pairs were taken from a cohort of 314 patients, which could be considered low. The size of the cohort could then be increased to augment the training database and refine the output of the model, optimizing AI training and reducing the chance of overfitting the model [20]. It is also worth mentioning that our algorithm did not have external validation as it is a first-phase development, and testing the finalized model on independent data is necessary to draw more specific conclusions on its clinical impact. Additionally, the ability of cardiac devices to differentiate arrhythmia types should be considered when using the extracted data to train the model [21,22]. On this matter, our model did not distinguish among AF, atrial tachycardia, and atrial high-rate episodes, making it hard to fully characterize the clinical relevance of the algorithm. Further research is being conducted considering minimal times, ECG information, and heart rate to fully characterize arrhythmia type. Finally, we extracted the data from devices from the same vendor, and we cannot affirm that this model can be reproducible with other commercial brands. However, fine-tuning methods can be used to train the algorithm using information from other devices. Taking all the aforementioned factors into consideration, further research could focus on increasing the prediction period and on differentiating types of arrhythmias given the clinical implications of de novo episodes of AF and the need for anticoagulation therapy.

Finally, we would like to mention the importance of an intuitive web design to present the obtained data. It has been proven that the use of mobile apps in health care is determined by how user-friendly and intuitive they are and the amount of data that must be input by users (eg, health care professionals) for the apps to function (eg, patient demographics, clinical variables, or diagnostic data) as a higher data entry burden may negatively affect usability and adoption [23]. If an application quickly displays the prediction along with the patient’s arrhythmia risk percentage, it is more likely that health care professionals will use it.

In conclusion, this study provides preliminary evidence that changes in arrhythmic burden may be estimated with limited but measurable sensitivity and specificity using data derived from implantable cardiac devices. A graphical overview of the study and its main findings is provided in Multimedia Appendix 8 (Visual Abstract). The predictive performance of the model may improve with further development and validation in larger cohorts that include additional demographic and clinical variables. These elements, together with the user-friendly visualization of predictions in a simple application, suggest that this initial phase of the study may serve as a basis for future research. Further studies are warranted to determine the clinical utility of this approach for the monitoring and management of patients with implantable cardiac pacing devices.

## Supplementary material

10.2196/85841 Multimedia Appendix 1 Inclusion criteria and patient recruitment.

10.2196/85841 Multimedia Appendix 2 Selection of input and output data.

10.2196/85841 Multimedia Appendix 3 Data volume and preprocessing.

10.2196/85841 Multimedia Appendix 4 Artificial intelligence–based prediction model.

10.2196/85841 Multimedia Appendix 5 Shapley Additive Explanations feature importance.

10.2196/85841 Multimedia Appendix 6 Confidence estimation.

10.2196/85841 Multimedia Appendix 7 Comparison of our model to other predictive models.

10.2196/85841 Multimedia Appendix 8 AI for the Prediction of Atrial Fibrillation or Atrial Tachycardia Episodes in Patients With Pacemakers.

## Abbreviations

AF: atrial fibrillation
AI: artificial intelligence
ECG: electrocardiogram
ML: machine learning
SHAP: Shapley additive explanations
