# Supplementary material for: Short-Term Arrhythmia Prediction Using AI Based on Daily Data From Implantable Devices: Multicenter Prospective Observational Study
Source: JMIR Cardio. 2026 Mar 18;10:e85841. doi: 10.2196/85841 (PMC12998600; doi:10.2196/85841)
Supplement: Multimedia Appendix 1 [file cardio-v10-e85841-s001.docx]

## Multimedia Appendix 1: Inclusion criteria and patient recruitment

Inclusion criteria for the study were:

- Age over 18 years.
- Indication for first implantation or replacement of a DDD or CRT-P pacemaker.
- Signed informed consent obtained prior to enrollment.

Exclusion criteria were:

- Patients undergoing renal hemodialysis.
- Permanent AF.
- History of heart transplantation or high likelihood of transplantation during the study followup.
- Pregnancy.
- Inability or unwillingness to complete follow-up visits.
- Life expectancy of less than one year.

A total of 763 patients were recruited across the different participating centers, shown in Table A.1.

Table A.1: Patients recruited in each of the participating centers.

| **Center** | **Patients** |
| --- | --- |
| HU DE BADAJOZ | 130 |
| HCU LOZANO BLESA | 125 |
| HUC SAN CARLOS | 87 |
| HU 12 DE OCTUBRE | 85 |
| HU PUERTA DE HIERRO | 82 |
| HU DE BURGOS | 80 |
| CLINICA LA LUZ | 68 |
| HCU DE SALAMANCA | 44 |
| HU QUIRONSALUD MADRID | 38 |
| HU INFANTA LEONOR | 11 |
| CLINICA MONTPELLIER, GRUPO HLA | 10 |
| H VIRGEN DE LA CONCHA | 3 |

To get the best results for AI training, patients with high amounts of missing data (mostly due to problems during the communication between the pacemaker and the central server) were excluded. Patients with less than 45 days of measurements are also excluded since they can’t be used by the algorithm. The remaining 314 patients were used for the final analysis.
