# Supplementary material for: Short-Term Arrhythmia Prediction Using AI Based on Daily Data From Implantable Devices: Multicenter Prospective Observational Study
Source: JMIR Cardio. 2026 Mar 18;10:e85841. doi: 10.2196/85841 (PMC12998600; doi:10.2196/85841)
Supplement: Multimedia Appendix 2 [file cardio-v10-e85841-s002.docx]

## Multimedia Appendix 2: Selection of Input and Output Data

Since not all patients had the same number of days recorded, the data were divided into 31-day observation sequences, which were used as input data. The output was initially defined as a classification based on the difference in the average number of arrhythmias between the 31-day observation period and the following 14 days (which the model attempts to predict), categorized as:

- **Increase in arrhythmias:** The average number of arrhythmias increased.
- **Stable:** The average number of arrhythmias remained unchanged.
- **Decrease in arrhythmias:** The patient had arrhythmias, but the average number decreased.

Later, we also decided to distinguish between patients who had arrhythmias during the observation period and those who did not. The data were separated depending on whether arrhythmias were present during the initial 31 days, resulting in four final classes: “Increase”, “Decrease or Stable”, “Increase from 0” (new onset of arrhythmias), and “Remains at 0”. An example of this classification can be seen in Figure 3.


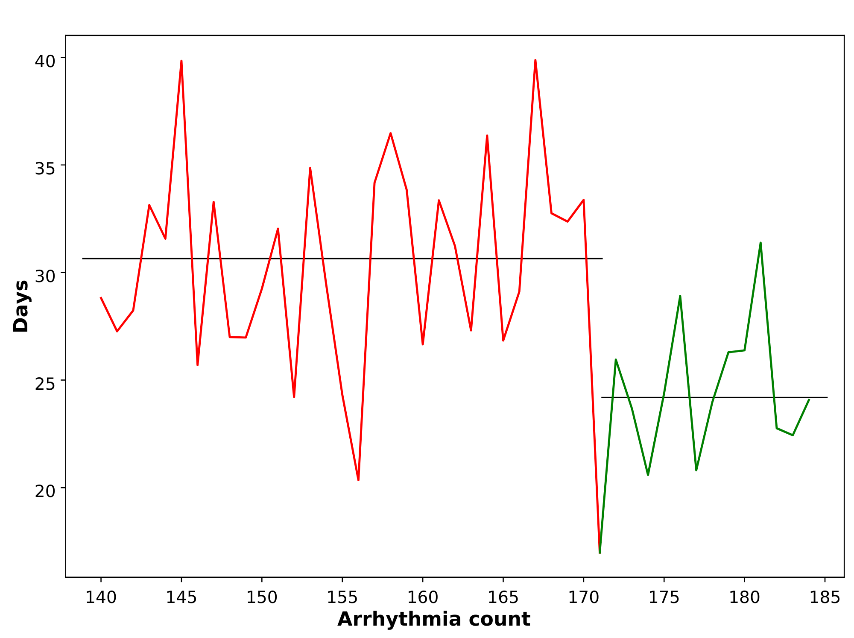


Figure 3: Example of the classification of a patient with decreasing arrhythmias. The red section indicates the 31-day observation period, and the green section corresponds to the following 14 days. The solid lines show the average number of arrhythmias in each period.
