# Supplementary material for: Short-Term Arrhythmia Prediction Using AI Based on Daily Data From Implantable Devices: Multicenter Prospective Observational Study
Source: JMIR Cardio. 2026 Mar 18;10:e85841. doi: 10.2196/85841 (PMC12998600; doi:10.2196/85841)
Supplement: Multimedia Appendix 3 [file cardio-v10-e85841-s003.docx]

## Multimedia Appendix 3: Data Volume and Preprocessing

First, all data were converted to a common format and error codes were removed. Missing data, whether due to recording errors or pacemaker models lacking certain variables, were imputed using strategies specific to each variable type:

Missing values for arrhythmia count or duration were assumed to be zero.

For missing values in the percentage of left ventricular pacing:

If no value was recorded on any day, it was assumed that the pacemaker did not support this type of pacing, and the value was set to 0.

If the variable appeared on some days but not others, missing values were filled using the last valid previous value; if unavailable, the next available value was used.

For missing values in ventricular rate during atrial tachyarrhythmia (RTA), values were set to 0 if no arrhythmia was present on that day. Otherwise, the mean of the available values from other days with arrhythmia was used.

For any other variable:

If missing, the value from the previous day was used; if unavailable, the next valid value was used.

If no valid values were available before or after, the overall mean across all patients was used for that variable.

After preprocessing, the data were divided into 45-day sequences (31 days of observation and 14 days for prediction), yielding a total of 65,243 data sequences:

Patients without arrhythmias who remained at 0: 45,937 (70.4%)

Patients without arrhythmias who developed new episodes: 3,765 (5.8%)

Patients with arrhythmias whose episodes decreased or remained stable: 11,029 (16.9%)

Patients with arrhythmias whose episodes increased: 4,512 (6.9%)

To avoid bias in which an AI model could simply predict the majority class (e.g., always classifying “remains at 0” for patients without arrhythmias and “decreases or remains stable” for those with arrhythmias, achieving up to 87% accuracy but ignoring meaningful patterns), we experimented with several data balancing strategies. Ultimately, we opted to modify the loss function using a Balanced Cross Entropy approach to assign greater weight to underrepresented classes. This strategy improved performance while avoiding excessively long training times.
