# Supplementary material for: Short-Term Arrhythmia Prediction Using AI Based on Daily Data From Implantable Devices: Multicenter Prospective Observational Study
Source: JMIR Cardio. 2026 Mar 18;10:e85841. doi: 10.2196/85841 (PMC12998600; doi:10.2196/85841)
Supplement: Multimedia Appendix 4 [file cardio-v10-e85841-s004.docx]

## Multimedia Appendix 4: AI-Based Prediction Model

A one-dimensional convolutional neural network (1D-CNN) was trained, as this architecture allows the extraction of temporal evolution patterns of each variable, treating them as time-series signals. To reduce the risk of overfitting and prevent the network from memorizing spurious patterns, a dropout rate of 30% was applied during training, randomly deactivating neurons to encourage generalization.

The dataset was split into training, validation, and test sets, using 75%, 10%, and 15% of the total data, respectively. The validation set was used for model selection via early stopping, while the test set provided an unbiased estimate of final model performance on unseen data.

The model was trained using the Adam optimizer with a learning rate of 0.0005 and a batch size of 128. The final model achieved a classification accuracy of 78% on the test set, with an F1 score of 80%. Notably, accuracy was higher for the most represented classes, suggesting that model performance could improve further by increasing the number of training samples for underrepresented categories — for instance, by incorporating more patients whose condition worsens over time.

Results of the trained model can be seen as a confusion matrix in Figure 5.

**
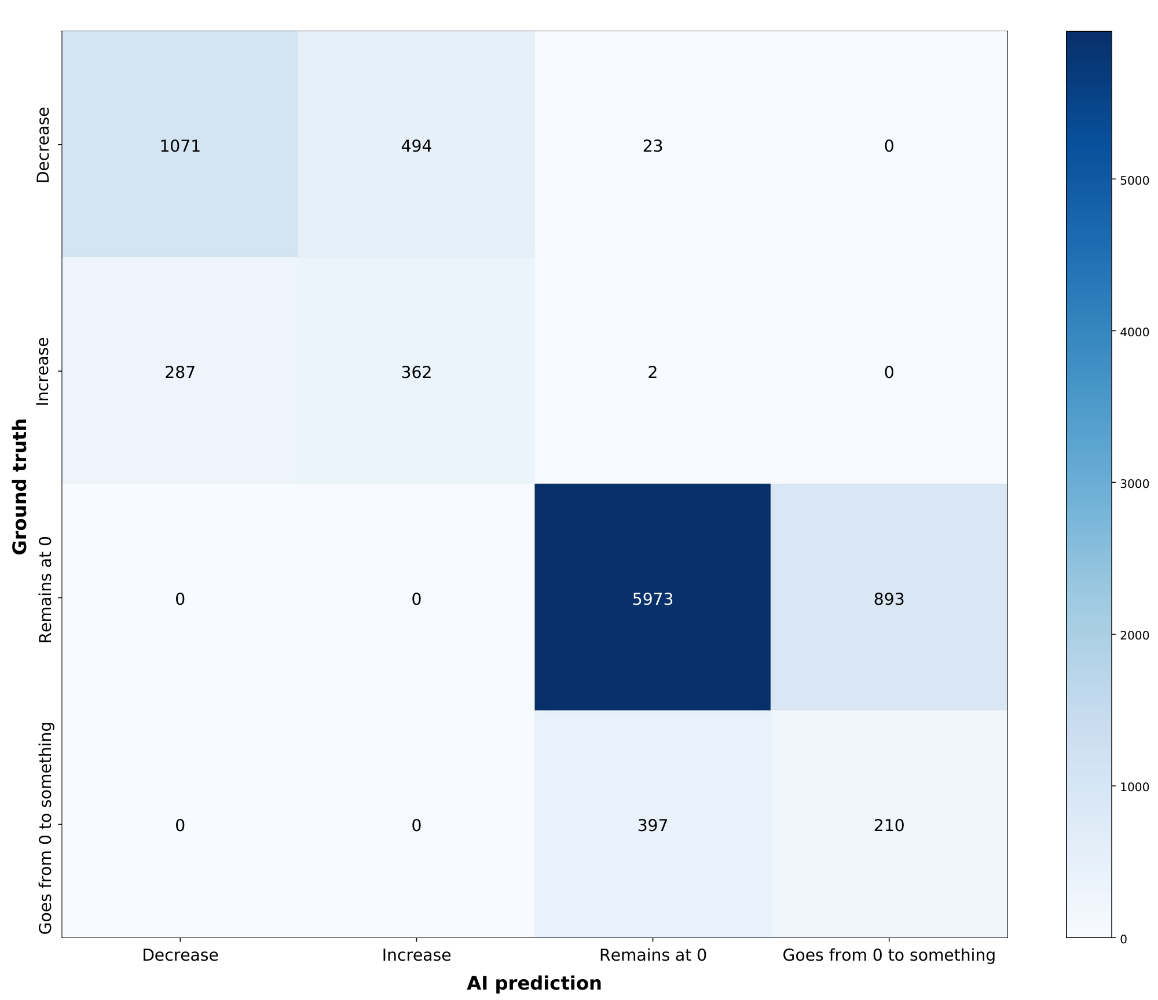
**

Figure 4: Confusion matrix of the trained model.
