# Supplementary material for: Short-Term Arrhythmia Prediction Using AI Based on Daily Data From Implantable Devices: Multicenter Prospective Observational Study
Source: JMIR Cardio. 2026 Mar 18;10:e85841. doi: 10.2196/85841 (PMC12998600; doi:10.2196/85841)
Supplement: Multimedia Appendix 5 [file cardio-v10-e85841-s005.docx]

## Multimedia Appendix 5: SHAP’s feature importance

SHAP is a game theory-based strategy to measure the contribution of each variable over the decision making of a prediction. Figure 5 depicts the mean results of SHAP over all the patients, colored by the final prediction.


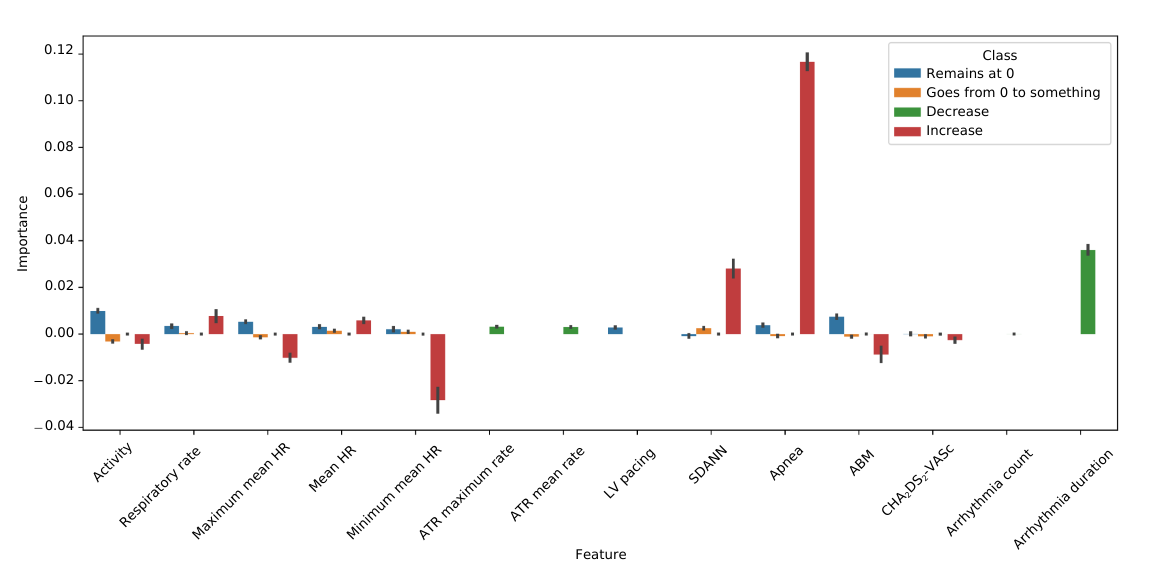


Figure 5: Variables used in the predictive model and estimated contribution to the decision-making of the predictions (positive values mean that higher values are associated with the class while negative values mean that lower values are associated with the class).
