# Supplementary material for: Short-Term Arrhythmia Prediction Using AI Based on Daily Data From Implantable Devices: Multicenter Prospective Observational Study
Source: JMIR Cardio. 2026 Mar 18;10:e85841. doi: 10.2196/85841 (PMC12998600; doi:10.2196/85841)
Supplement: Multimedia Appendix 6 [file cardio-v10-e85841-s006.docx]

## Multimedia Appendix 6: Confidence estimation

In addition to the SHAP framework described previously (Figure 5), we also analyzed the entropy of the model’s output probabilities to estimate prediction confidence. We observed that the entropy was significantly higher for incorrect predictions compared to correct ones.

Based on this observation, we trained a logistic regression model to estimate the likelihood of a correct prediction using the entropy value as input. This post-hoc model provides a probability score for prediction reliability, enabling clinical users to interpret the system’s confidence in each individual forecast. This model can be seen in Figure 6.


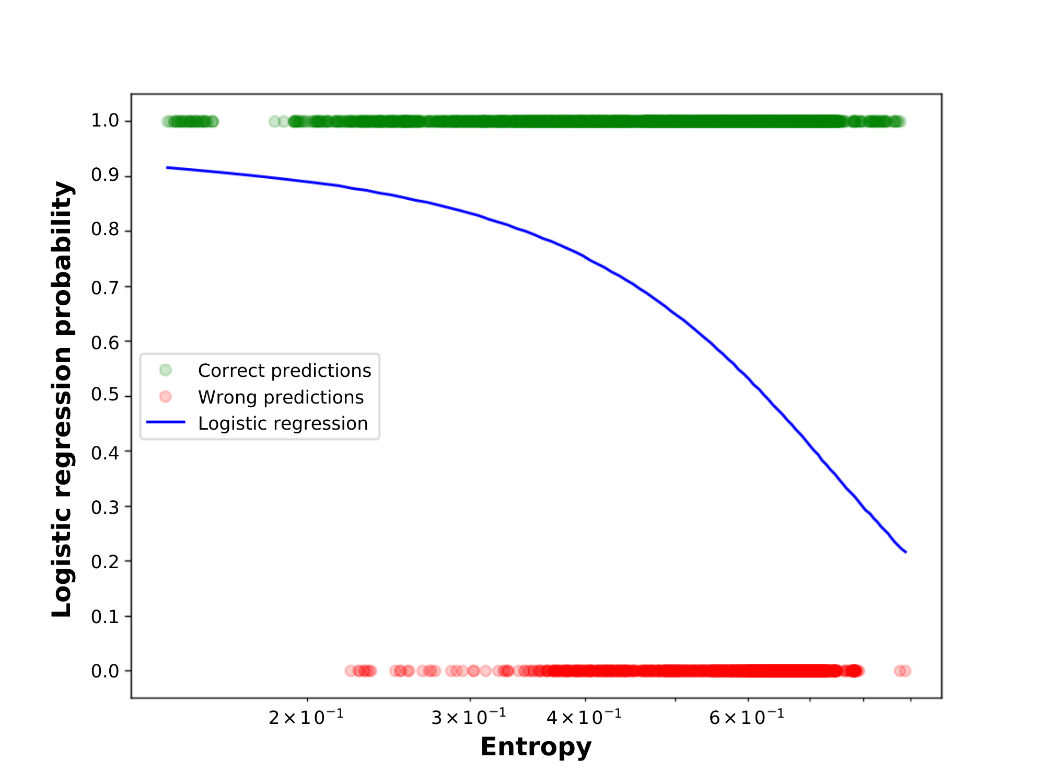


Figure 6: Results of entropy’s logistic regression training.
