# Supplementary material for: Short-Term Arrhythmia Prediction Using AI Based on Daily Data From Implantable Devices: Multicenter Prospective Observational Study
Source: JMIR Cardio. 2026 Mar 18;10:e85841. doi: 10.2196/85841 (PMC12998600; doi:10.2196/85841)
Supplement: Multimedia Appendix 7 [file cardio-v10-e85841-s007.docx]

## Multimedia Appendix 7: Comparison of our model to other predictive models

| **Models to predict AF comparison** | | | | | |
| --- | --- | --- | --- | --- | --- |
|  | **N** | **Information used to predict AF** | **Data time collection (or AI training time) – AF onset prediction period** | **Sensitivity** | **Specificity** |
| **Predictive models from clinical records** | | | | | |
| Kao et al, 2017 [1] | 2138 patients | Clinical records (no ECG) | 3 year medical information – prediction of AF onset within 1 year | 98.7 | NR |
| Tiwari et al., 2020 [2] | 2,252,219 records | 200 features from clinical records from 2011 to 2018 | prediction of AF onset within 6 months | 75 | 85 |
| Nadarajah et al, 2023 [3] | Not specified | clinical records from 1998 to 2008 | At least 1 year follow up – prediction of AF onset within 6 months | 78.1 | 73.1 |
| **Pedictive models from other sources of data** | | | | | |
| Gregoire et al, 2025 [4] | 872 patients | Holter recordings | 1 hour of each recording | 83.0 | 86.6 |
|  |  |  |  |  |  |
|  |  |  |  |  |  |
|  |  |  |  |  |  |
| Our study | 314 patients | Pacemakers (telemetry) | 31 days | 66.4 | 77.4 |

NR: not reported

**References**

1. Kao YT, Huang CY, Fang YA, Liu JC, Chang TH. Machine learning-based prediction of atrial fibrillation risk using electronic medical records in older aged patients. Am J Cardiol. Jul 1, 2023;198:56-63. [doi: 10.1016/j.amjcard.2023.03. 035] [Medline: 37209529]
2. Tiwari P, Colborn KL, Smith DE, Xing F, Ghosh D, Rosenberg MA. Assessment of a machine learning model applied to harmonized electronic health record data for the prediction of incident atrial fibrillation. JAMA Netw Open. Jan 3, 2020;3(1):e1919396. [doi: 10.1001/jamanetworkopen.2019.19396] [Medline: 31951272]
3. Nadarajah R, Wu J, Hogg D, et al. Prediction of short-term atrial fibrillation risk using primary care electronic health records. Heart. Jun 26, 2023;109(14):1072-1079. [doi: 10.1136/heartjnl-2022-322076] [Medline: 36759177]
4. Grégoire JM, Gilon C, Marelli F, et al. Short-term atrial fibrillation onset prediction using machine learning. Eur Heart J Digit Health. Sep 11, 2025;6(6):1159-1168. [doi: 10.1093/ehjdh/ztaf104] [Medline: 41267849]
