# Supplementary figures and images for: Short-Term Arrhythmia Prediction Using AI Based on Daily Data From Implantable Devices: Multicenter Prospective Observational Study
Source: JMIR Cardio. 2026 Mar 18;10:e85841. doi: 10.2196/85841 (PMC12998600; doi:10.2196/85841)

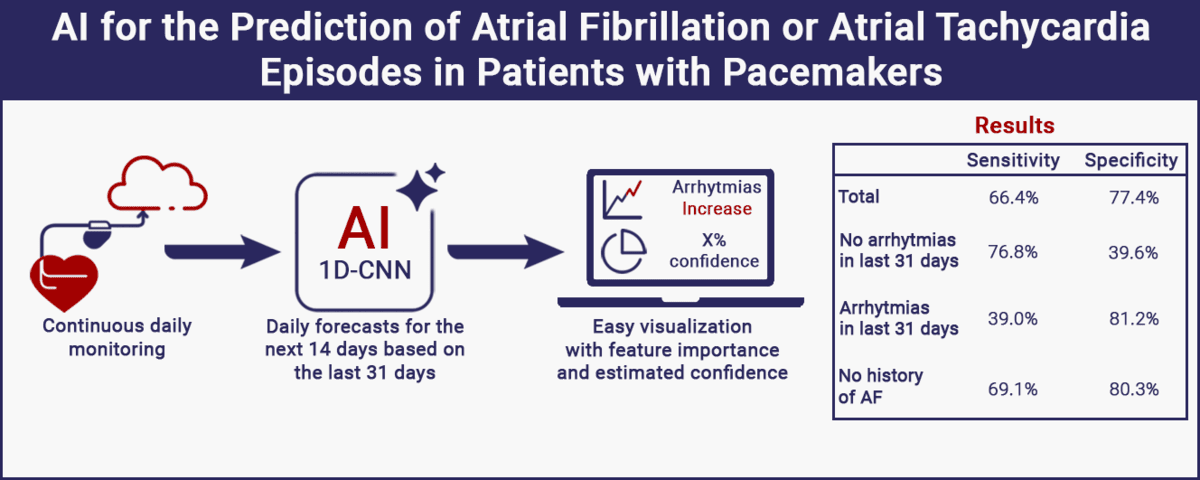

Supplement: Multimedia Appendix 8 [file cardio-v10-e85841-s008.png]
